# Generalized Spin–Curl Force Beyond the Stress Tensor

**DOI:** 10.3390/s25175367

**Published:** 2025-08-30

**Authors:** Tongtong Zhu, Guodong Zhu, Chuang Li, Bojian Shi, Rui Feng, Yongyin Cao, Yurui Fang, Weiqiang Ding

**Affiliations:** 1 School of Physics, Dalian University of Technology, Dalian 116024, China; zhutongtong@dlut.edu.cn (T.Z.);; 2 College of Metrology Measurement and Instrument, China Jiliang University, Hangzhou 310018, China; 3 Institute of Advanced Photonics, School of Physics, Harbin Institute of Technology, Harbin 150001, China; 4 Collaborative Innovation Center of Extreme Optics, Shanxi University, Taiyuan 030006, China

**Keywords:** optical force, optical manipulation, optical sensing

## Abstract

The optical force exerted on a dipole particle can be divided into gradient force, scattering force, and spin–curl force, all of which can be derived from Maxwell’s stress tensor with the dipole approximation. Here, we identify an additional spin–curl force for arbitrary objects beyond the dipole approximation, which is named the generalized spin–curl force in this paper. The generalized spin–curl force originates from the Minkowski force density and depends on the imaginary parts of the permittivity, permeability, and chirality of the object. However, it remains imperceptible in conventional optical force calculations due to its exact cancellation by a compensatory surface force during MST surface integration. The study of the generalized spin–curl force provides critical insights into elucidating the mechanisms underlying optical momentum transfer and internal force distribution within complex media. Furthermore, the generalized spin–curl force offers a novel mechanism for enhancing optical sensors, enabling highly sensitive detection of absorptive or chiral perturbations in systems such as microcavities and metasurfaces. Its ability to manipulate internal force distributions also provides new pathways for advancing optical force probes and chirality-selective sensing at the nanoscale.

## 1. Introduction

Optical forces play a pivotal role in modern science and technology [1,2,3], including optical manipulation [4,5,6], chirality sorting [7,8], optical spanners and stretchers [9], thermally limited force sensing [10], and biological systems [11,12,13]. In all this research, the optical force calculation is prominent and is primarily achieved through the integration of Maxwell’s stress tensor, which is derived from the Lorentz force densities and Maxwell’s equations [14,15,16]. When media are introduced, several different formulations of force density have been proposed, with the most renowned ones by Einstein–Laub, Ampère–Lorentz, Minkowski, and Abraham [14,17,18,19]. From a physical perspective, Ampère and Lorentz posited that the electric and magnetic fields exert forces on the densities of total charges and currents [14], based on which the Ampère–Lorentz force density was proposed. Interestingly, several studies have questioned the accuracy of the Ampère–Lorentz force density in recent years, but subsequent investigations have refuted these claims [20,21,22,23,24].

Corresponding to the force densities, various stress tensors have also been proposed, with the Minkowski stress tensor (MST) being the most commonly used in optical force calculations [21]. By employing the MST and dipole approximation, an additional scattering force from the curl of the spin angular momentum of a light field has been identified and studied [25,26]. Up to now, it is regarded that the optical force calculated from the surface integration of MST is the total optical force. It certainly contains all the contributions of the gradient force, scattering force, and the spin–curl force in the case of dipole approximation.

The identification of the generalized spin–curl force (GSCF) enriches the understanding of light–matter interactions in complex media, which is essential for designing highprecision optical force sensors. The GSCF, though canceled in net force measurements, may play a critical role in redistributing internal forces within sensor structures such as photonic crystal cavities or metasurfaces, potentially enhancing light–matter interactions and enabling new sensing modalities [10,27]. In optical sensors, GSCF-driven internal force redistribution may enable new detection paradigms: for instance, in whispering-gallerymode biosensors, absorption-induced GSCF perturbations could amplify single-molecule binding signals; in terahertz chiral sensors, spin–curl interactions might enhance the discrimination of enantiomers via momentum transfer [28]. Additionally, the ability to probe internal force distributions non-invasively could lead to novel sensor designs for biomedical applications, such as intracellular force mapping or nanomechanical property characterization, and the chirality-sensitive nature of GSCF may be harnessed in label-free biosensors for discriminating biomolecular handedness [29].

In this work, we checked the MST in the most general case of medium and cautiously identified an additional term in force density beyond the MST, which consequently contributes an additional term in the optical force. The new term arises from the spin angular momentum of the total field and depends on the imaginary parts of the permittivity, permeability, and chirality of the object. However, the additional force is imperceptible, due to its cancellation by a surface force. The properties and potential applications of the addition force are discussed, with particular emphasis on its implications for and critical role in advanced optical sensing technologies.

## 2. Theory

The Lorentz force exerted on charged matter is given by

(1) f=ρE+J×B,

where E and B are the electric field and the magnetic induction, respectively; ρ and J are the densities of the charge and current, respectively. Employing the Maxwell’s equations in matter, the force density becomes

$$\begin{array}{cc} f= & \left(\nabla \cdot D\right)E-D\times \left(\nabla \times E\right)+\left(\nabla \cdot B\right)H \end{array}$$

(2) $$\begin{array}{cc} \; & -B\times \left(\nabla \times H\right)-\frac{\partial }{\partial t}\left(D\times B\right) \end{array}$$

(3) $$\begin{array}{cc} \; & =\nabla \cdot {\overset{↔}{T}}_{M}-\frac{\partial }{\partial t}\left(D\times B\right), \end{array}$$

where ${\overset{↔}{T}}_{M}$ is the Minkowski stress tensor of

(4) $${\overset{↔}{T}}_{M}=\left(DE\right)+\left(BH\right)-\frac{1}{2}\overset{↔}{I}\left(D\cdot E+B\cdot H\right).$$

In order to calculate the averaged optical force, a time-average operation should be performed to neglect the effect of fast oscillation of the optical fields, and the last term in Equation (3) is zero. Without loss of generality, we assume that the time-varying fields are time-harmonic monochromatic plane waves,

(5) Er,t=ReEcexp(−iωt),Br,t=ReBcexp(−iωt).

Then, the time-averaged Minkowski force density is given by

(6) $$\langle f\rangle =\nabla \cdot \langle {\overset{↔}{T}}_{M}\rangle$$

with the time-averaged Minkowski stress tensor of

(7) T↔M=12ReDcEc*+12ReBcHc*−14ReI↔Dc·Ec*+Bc·Hc*.

Then, the total optical force can be obtained through the integration of the Minkowski force density

(8) $$\begin{array}{c} \langle F_{M}\rangle =\int_{V}\nabla \cdot \langle {\overset{↔}{T}}_{M}\rangle dV=\oint_{S}\langle {\overset{↔}{T}}_{M}\rangle \cdot \hat{n}ds, \end{array}$$

where *V* is the volume of the object, and $\hat{n}$ is the normal vector of its surface *S*. This is the widely known procedure to derive the optical force and the stress tensor, which can be found in electrodynamics-related textbooks [30,31,32].

Here, we argue that, in the most general cases, there should be another item in the total optical force calculation. As we know, it is quite vague in the derivation from Equation (2) to Equation (3) [32]. The degenerate form of the Minkowski stress tensor under vacuum conditions can be derived by using the formula [30]

(9) $$\nabla \left(E\cdot E\right)=2\left(E\cdot \nabla \right)E+2E\times \left(\nabla \times E\right).$$

In the derivation from Equation (2) to Equation (3), the following formula could be used, which is

(10) $$\nabla \left(D\cdot E\right)=2\left(D\cdot \nabla \right)E+2D\times \left(\nabla \times E\right).$$

However, this formula only holds for homogeneous, isotropic, and lossless medium. Mathematically, the general relation for a complex medium is [33]

(11) $$\begin{array}{cc} \nabla \left(D\cdot E\right)= & D\times \left(\nabla \times E\right)+E\times \left(\nabla \times D\right)+\left(D\cdot \nabla \right)E+\left(E\cdot \nabla \right)D. \end{array}$$

For a simple medium, Equations (10) and (11) become exactly the same. In a general complex medium, however, they may give different results.

Employing the more general Equation (11), we reexamine the Minkowski force density for the time-harmonic fields and obtain

(12) f′=∇·T↔M+12Re∇×Dc×Ec*+12Re∇×Bc×Hc*.

The last two items are not necessarily zero in general cases. The forms of the two nonzero items indicate that they are related to the angular momentum of the electric field and magnetic field. Consequently, the optical force for the generalized case can be calculated as

(13) Ftot=∫V∇·T↔MdV+∫V12Re∇×Dc×Ec*dV   +∫V12Re∇×Bc×Hc*dV=FM+FSurface+FGSCF,

where

(14) FSurface=12∫S+{Re∇×Dc×Ec*+Re∇×Bc×Hc*}dS−12∫S−{Re∇×Dc×Ec*+Re∇×Bc×Hc*}dS

is the surface force with the inner and outer surfaces $S^{+}$ and $S^{-}$, respectively. The additional body force is given by

(15) FGSCF=∫V−{12Re∇×Dc×Ec*           +12Re∇×Bc×Hc*}dV.

Since $E_{c}\times E_{c}^{*}$ and $H_{c}\times H_{c}^{*}$ are related to the angular momentum density of the electric field E and the magnetic field H [34,35], respectively, the additional force can be interpreted as the generalized spin–curl force (GSCF). It is emphasized that the GSCF described in Equation (15) is distinct from the spin–curl force discussed in Refs. [25,26], which arises solely from the spin angular momentum of the electric field. More importantly, the GSCF is not included in the integration of MST. It should be noted that the sum of the surface force and the GSCF is zero, i.e., $\langle F_{\mathrm{Surface}}\rangle +\langle F_{\mathrm{GSCF}}\rangle =0$. As a result, the generalized optical force $\langle F_{\mathrm{tot}}\rangle$ in Equation (13) is reduced to $\langle F_{M}\rangle$, i.e.,

(16) $$\langle F_{\mathrm{tot}}\rangle =\langle F_{M}\rangle .$$

Without loss of generality, we consider a chiral particle with complex permittivity and permeability, i.e.,

(17) $$\begin{array}{cc} D_{c}= & \varepsilon E_{c}+\frac{i\kappa }{c}H_{c},\\ B_{c}= & \mu H_{c}-\frac{i\kappa }{c}E_{c}, \end{array}$$

where κ is the chiral parameter. Then, the additional force becomes

(18) $$\langle F_{\mathrm{GSCF}}\rangle =\langle F_{\mathrm{GSCF}}^{E}\rangle +\langle F_{\mathrm{GSCF}}^{H}\rangle ,$$

with

(19) FGSCFE=12∫V{Re∇×εEc×Ec*−Imκc∇×ReHc×Ec*−Reκc∇×ImHc×Ec*}dV,

(20) FGSCFH=12∫V{Re∇×μHc×Hc*+Imκc∇×ReEc×Hc*+Reκc∇×ImEc×Hc*}dV.

Using the identity A×B=−B×A for vectors, a further simplification yields

(21) FGSCF=12∫V{Re∇×εEc×Ec*+Re∇×μHc×Hc*+2Imκc∇×ReEc×Hc*}dV.

In Equation (21), the first two terms are related to the spins of the electromagnetic field, and the last term depends on the chirality. Since $E_{c}\times E_{c}^{*}$ and $H_{c}\times H_{c}^{*}$ are pure imaginary, the first two terms are nonzero in the case of ε and μ with nonzero imaginary parts. Similarly, the last term is nonzero if the imaginary part of κ is nonzero.

## 3. Numerical Results and Discussion

**Figure 1:**
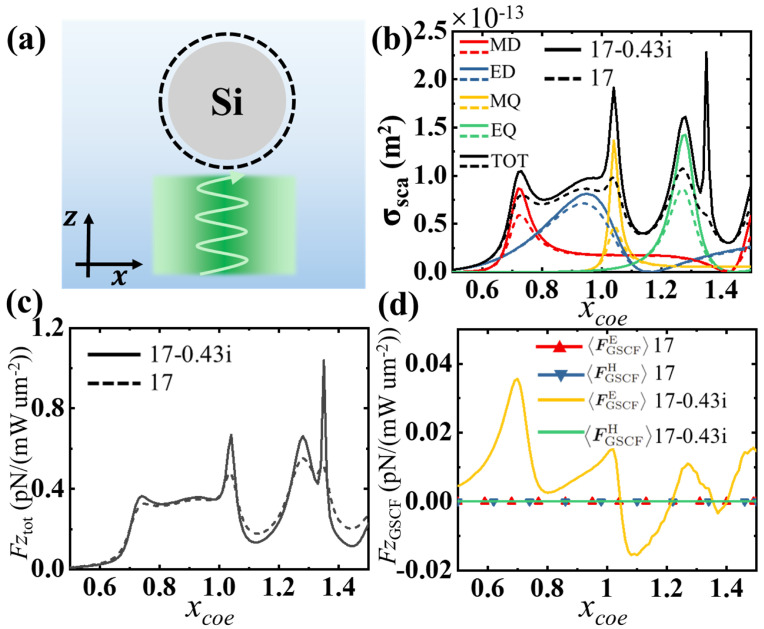
(**a**) Schematic of structure. A homogeneous dielectric sphere, such as silicon, with a manually tunable imaginary permittivity is immersed in water. The incident light is a linearly polarized green light with the wavelength of 532 nm. (**b**) Scattering cross section and multipolar decomposition spectra of the spheres with permittivity of ε=17 (dashed curves) and ε=17−0.43i (solid curves), respectively. The red, blue, yellow, and green lines correspond to the scattering of magnetic dipoles (MD), electric dipoles (ED), magnetic quadrupoles (MQ), and electric quadrupoles (EQ), respectively. (**c**) The z-component of the total optical force (Equation (13)) corresponds to the spheres with ε=17 and ε=17−0.43i, respectively. (**d**) The GSCF associated with the spin angular momentum of the electric and magnetic fields with ε=17 and ε=17−0.43i, respectively.

In this section, we focus on and study the properties of the GSCF. We numerically calculate the optical response of a dielectric sphere illuminated by a laser beam with wavelength λ=532 nm, as shown in Figure 1. The dielectric particle (such as silicon) has a real part of permittivity of ℜ(ε)=17 and a manually tunable imaginary part of permittivity of ℑ(ε)=0 or −0.47 (for the comparison of the additional force item of the GSCF). The dielectric sphere is levitated in water with refractive index n=1.33, and the light is linearly polarized and propagates along the +z-axis. For simplicity, we introduce the normalized size parameter $x_{coe}=kr$ with the radius of the sphere *r* and the wave number of the light k=2π/λ. In the following discussion, we manually modify the parameters of the sphere, such as the imaginary part of ε and the chirality κ, in order to show the contribution of the GSCF. Figure 1b shows the scattering cross section of the sphere $\sigma_{\mathrm{sca}}$ as a function of the size parameter $x_{coe}$. The results show that, when the imaginary part of the permittivity is taken into account, the scattering of the spheres at the resonance becomes pronounced, resulting in a stronger optical force [Figure 1c, where we focus on the optical force along the z-axis only]. Figure 1d shows the GSCF as a function of $x_{coe}$. When the dielectric material is lossless, both the $\langle F_{\mathrm{GSCF}}^{E}\rangle$ and the $\langle F_{\mathrm{GSCF}}^{H}\rangle$ are zero, indicating that the generalized spin–curling force is imperceptible in traditional optical force calculations. When the imaginary part of the dielectric function is considered, the $\langle F_{\mathrm{GSCF}}^{H}\rangle$ remains zero because the magnetic permeability of the dielectric sphere is 1 at optical frequency, while the $\langle F_{\mathrm{GSCF}}^{E}\rangle$ exhibits a significant additional optical force in the z direction, which varies significantly with $x_{coe}$. This can be attributed to the imaginary part of $E_{c}\times E_{c}^{*}$ in Equation (19), which generates an additional total optical force $\langle F_{\mathrm{GSCF}}\rangle$ when the permittivity has a nonzero imaginary part. Mathematically speaking, this is because $E_{c}\times E_{c}^{*}$ and $H_{c}\times H_{c}^{*}$ are pure imaginary.

**Figure 2:**
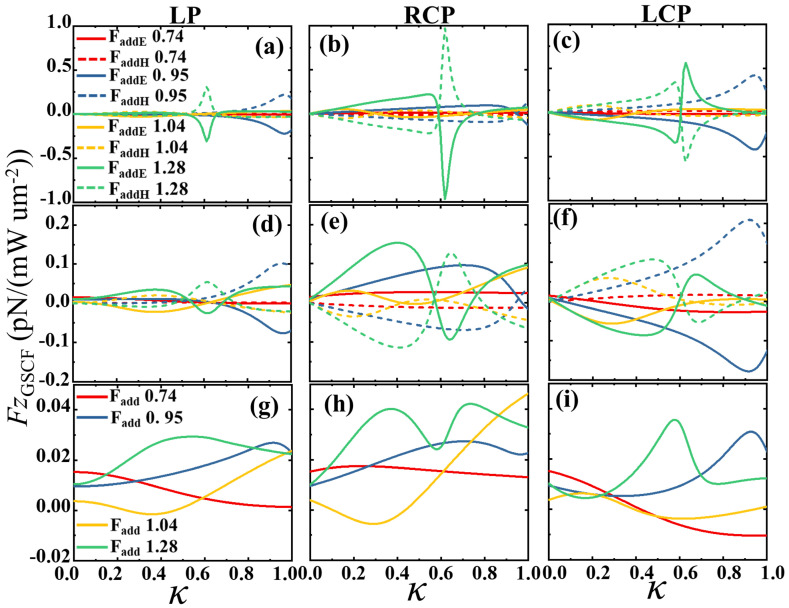
The GSCF forces on (**a**–**c**) non-absorbing chiral spheres (ε=17) and (**d**–**f**) on absorbing chiral spheres (ε=17−0.43i) under the linearly polarized (LP) and left/right-handed circularly polarized (LCP/RCP) light. The normalized radius of $x_{coe}=0.74$, 0.95, 1.04, and 1.28 are selected, which correspond to the magnetic dipoles (MD), electric dipoles (ED), magnetic quadrupoles (MQ), and electric quadrupoles (EQ), respectively (See Figure 1b). (**g**–**i**) The sum of forces corresponding to the spin angular momentum of the electric and magnetic in (**d**–**f**). The imaginary part of the chiralty is zero, Im(κ)=0, for all the figures.

Next, we consider the effect of chirality on the GSCF, i.e., κ≠0. Figure 2 illustrates the GSCF on a chiral silicon sphere at Mie resonance (ED, MD, EQ, MQ) for a real chirality parameter. It is observed that when a non-absorbing silicon sphere is illuminated by linearly polarized (LP) and left/right-handed circularly polarized (LCP/RCP) light [see Figure 2a–c], the electric and magnetic fields produce additional forces that contribute significantly to the total optical force. However, these forces cancel each other out because $\langle F_{\mathrm{GSCF}}^{E}\rangle$ and $\langle F_{\mathrm{GSCF}}^{H}\rangle$ have opposite signs, resulting in a net value of zero for the additional spin–curl force. Yet, when an absorbing silicon sphere (ε=17−0.43i) is illuminated by linearly and circularly polarized light [see Figure 2d–f], the additional forces generated by the electric and magnetic fields do not completely cancel each other out. Therefore, the net GSCF can be observed [see Figure 2d–f], and the GSCF at the Mie resonance changes significantly with the change in the real number κ, which is consistent with the third term of Equation (21), further verifying the theoretical results.

**Figure 3:**
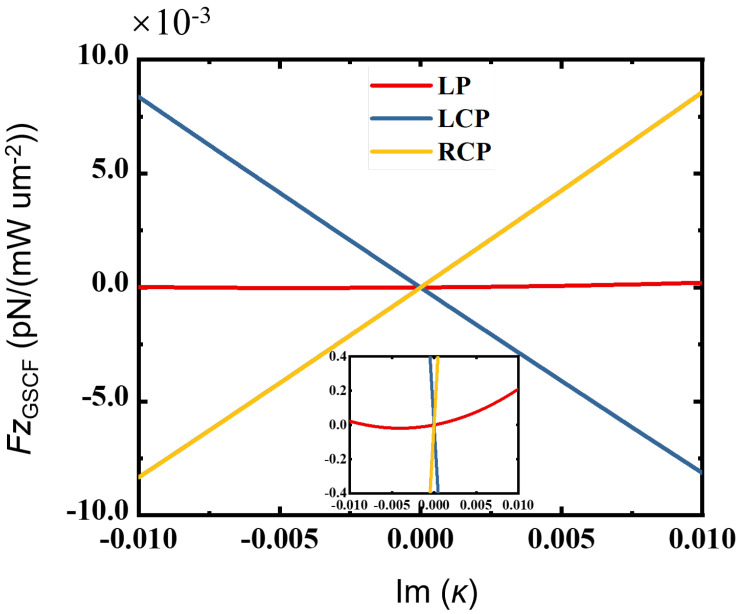
The GSCFs on a chiral sphere when illuminated by linearly polarized (LP), left/righthanded circularly polarized (LCP/RCP) light with a real permittivity, and a complex chiral parameter κ. The inset shows the details of the GSCF varying with the imaginary part of the chiral parameter for the linearly polarized light. The other parameters are $x_{coe}=0.74$, ε=17, and Re(κ)=0.01.

To further explore the regulatory effect of the chiral parameter κ and the permittivity ϵ on the GSCF, as shown in Figure 3, we plot the additional spin–curl force on the chiral silicon sphere illuminated by linearly polarized (LP) and left/right-handed circularly polarized (LCP/RCP) light, with real permittivity and a complex chiral parameter. It shows that the GSCF scales linearly with the imaginary part of κ under circularly polarized light, while it remains nearly constant under linearly polarized light. The inset of Figure 3 implies that the GSCF is nonzero for linearly polarized light because of the effects of the scattered field.

**Figure 4:**
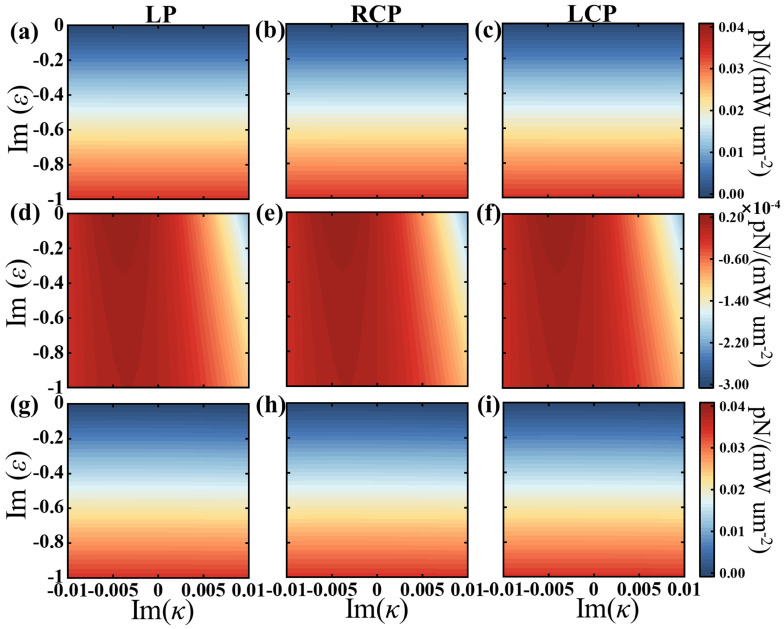
The GSCFs on a chiral sphere when illuminated by linearly polarized (LP), left/right-handed circularly polarized (LCP/RCP) light with a complex permittivity, and a complex chiral parameter κ. (**a**–**f**) show the parts of the additional force originating from the spin of the electromagnetic field and the chiral, respectively. (**g**–**i**) shows the total additional force. The other parameters are $x_{coe}=0.74$, Re(ε)=17, and Re(κ)=0.01.

Figure 4 shows the GSCF on a chiral silicon sphere illuminated by linearly polarized (LP), left/right-handed circularly polarized (LCP/RCP) light with complex permittivity and a complex chiral parameter. Figure 4a–f show the parts of the GSCF originating from the spin of the electromagnetic field and the chirality, respectively. Figure 4a–c show that the additional optical force $\langle F_{\mathrm{GSCF}}^{E}\rangle$ is essentially the same for different polarizations and increases with the imaginary part of permittivity of the chiral sphere. Because the magnetic permeability is a pure real number of 1, the additional force $\langle F_{\mathrm{GSCF}}^{H}\rangle$ in Figure 4d–f is primarily generated by electromagnetic field coupling. Figure 4g–i show the total GSCF. These show that the additional force is mainly caused by the spin. As shown above, all the numerical results are consistent with the theoretical results, further verifying the regulation of GSCF by chirality and dielectric function.

## 4. Conclusions

In summary, we have identified a generalized spin–curl force (GSCF) beyond the Minkowski stress tensor, which arises from the spin angular momentum of the electromagnetic fields and depends on the imaginary parts of the permittivity, the permeability, and the chiral parameter of the particle. Distinctly from the spin–curl force discussed in Refs. [25,26] at the case of dipole approximation, both the contributions from the electric field and magnetic field are identified for arbitrary objects and give the full origin of the spin–curl force of a light field. More importantly, the GSCF reported here is not included in the contribution of stress tensor integration. For nonabsorbing objects, the GSCF is always zero. However, for some resonant particles with proper imaginary parts of ε, μ and chirality κ, the amplitude of the GSCF may be comparable to the force calculated from the stress tensor integration. Though the GSCF is imperceptible due to its exact cancellation by a compensatory surface force during MST surface integration, the GSCF plays a pivotal role in critical insights into elucidating the mechanisms underlying optical momentum transfer [17,36] and internal force distribution [37,38,39] within complex media. In addition, the experimental detection of the generalized spin–curl force (GSCF) could be realized in integrated photonic or nanomechanical systems [40].

Moreover, the identification of the GSCF opens new possibilities for designing nextgeneration optical sensors and lays the groundwork for innovative sensor architectures. The explicit accounting of GSCF could inform the design of highly sensitive optical force probes capable of resolving nanoscale mechanical properties in absorbing or chiral environments, which would be invaluable in fields like nanomedicine, soft matter physics, and micro-robotics [10,27,28,29]. For example, in resonant microcavities or metasurfaces used for label-free biosensing, the internal force distribution influenced by GSCF could be exploited to improve the sensitivity to analyte concentration or chirality [27]. Furthermore, it suggests transformative applications—e.g., monitoring GSCF-mediated internal stress in specialty-fiber sensors for real-time analyte detection or designing nano-engineered probes where absorption-dependent spin–curl forces transduce biochemical interactions into mechanical readouts with high sensitivity [28,29]. Future work may explore active tuning of the GSCF via material engineering to realize highly integrated multi-parameter optical sensing platforms, and the integration of GSCF-aware models into simulation tools may accelerate the development of self-calibrating sensors, ultimately bridging the gap between theoretical electrodynamics and practical metrology.

## Data Availability

The data that support the findings of this study are available from the corresponding author upon reasonable request.
